# Supplementary material for: SERPINA3-ANKRD11-HDAC3 pathway induced aromatase inhibitor resistance in breast cancer can be reversed by HDAC3 inhibition
Source: Commun Biol. 2023 Jul 6;6:695. doi: 10.1038/s42003-023-05065-w (PMC10326080; doi:10.1038/s42003-023-05065-w)
Supplement: Supplementary file 2 — Supplementary Figures [file 42003_2023_5065_MOESM2_ESM.pdf]

SERPINA3-ANKRD11-HDAC3 pathway induced aromatase inhibitor resistance in breast cancer can be reversed by HDAC3 inhibition

Jing Zhou, Mengdi Zhu, Qi Wang, Yiyuan Deng, Nianqiu Liu, Yujie Liu and Qiang Liu

Supplementary Figures

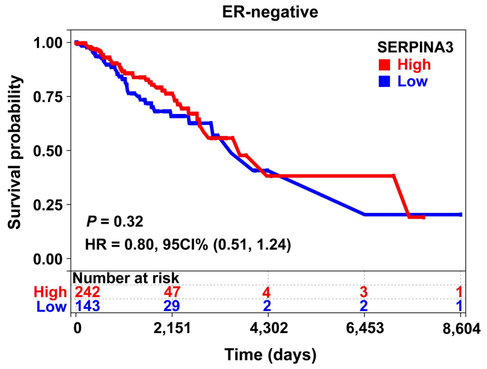

Supplementary Figure 1 Kaplan–Meier curves for overall survival of 373 ER- BC patients with high or low expression of SERPINA3. HR, hazard ratio; CI, confidence interval.

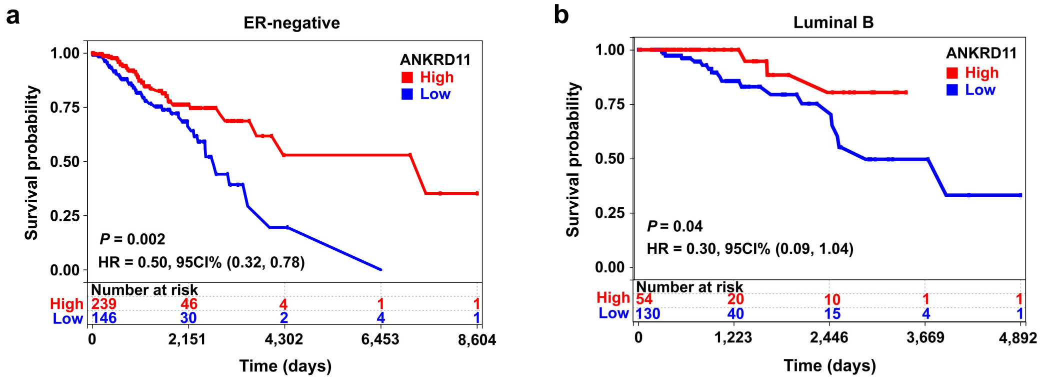

Supplementary Figure 2 Kaplan–Meier curves for overall survival of (a) 385 ER- BC patients and (b) 184 luminal B patients with high or low expression of ANKRD11. HR, hazard ratio; CI, confidence interval.

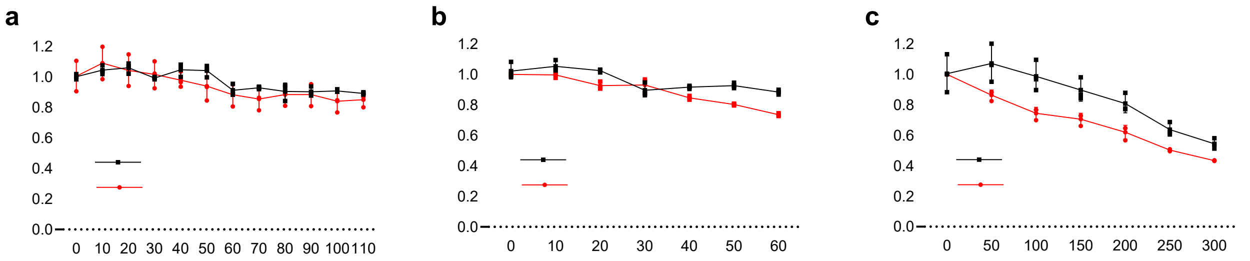

Supplementary Figure 3 ER+ BC cell line MCF-7 and T47D can not be remarkably inhibited by therapeutic blood concentrations of the aromatase inhibitors anastrozole (10-40 ng/ml), exemestane (10-20 ng/ml) and letrozole (50-100 ng/ml). MCF-7 and T47D cells were cultured with indicated doses of (a) anastrozole or (b) exemestane or (c) letrozole for 5 days, then MTT was performed. Data are representative of three independent experiments and presented as mean±SD.

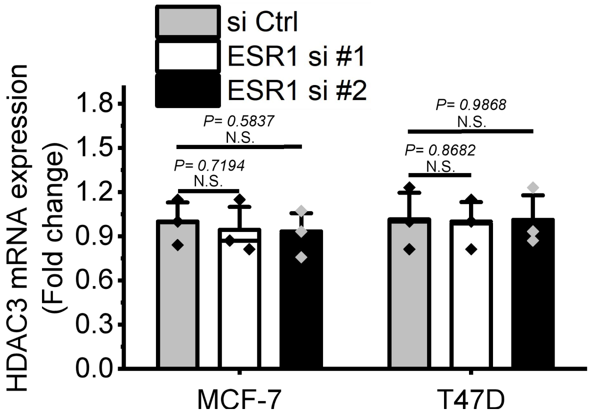

Supplementary Figure 4 QPCR results showed that ESR1 knockdown had no significant effect on HDAC3 expression in MCF-7 and T47D cells. Data are representative of three independent experiments and presented as mean±SD.

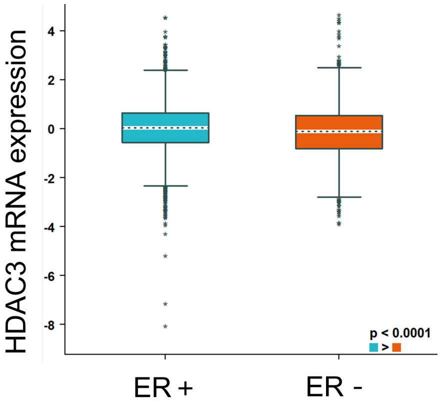

Supplementary Figure 5 Data retrieved from bc-GenExMiner v4.9 database indicates a slight decrease of HDAC3 in ER- BC compared with ER+ subtype. Data are representative of three independent experiments and presented as mean±SD.

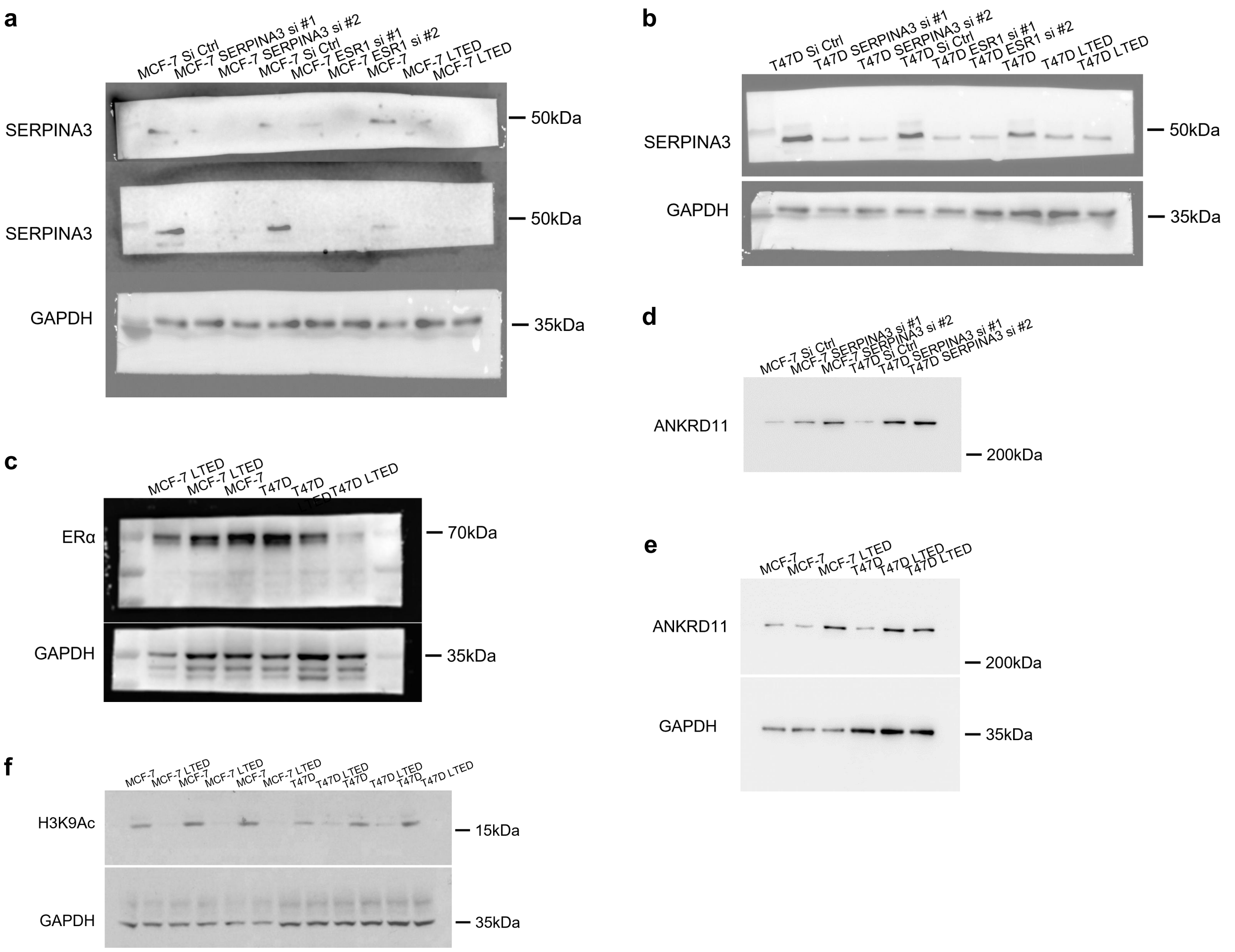

Supplementary Figure 6 The original uncropped images of western blotting of SERPINA3 and GAPDH in Fig. 2e, 3f and 4e in (a) MCF-7 and (b) T47D cells; (c) ERα and GAPDH in Fig. 3d; and ANKRD11 and GAPDH in (d) Fig. 4e and (e) Fig. 4g; (f) acetylated histone H3K9 and GAPDH in Fig. 5a.

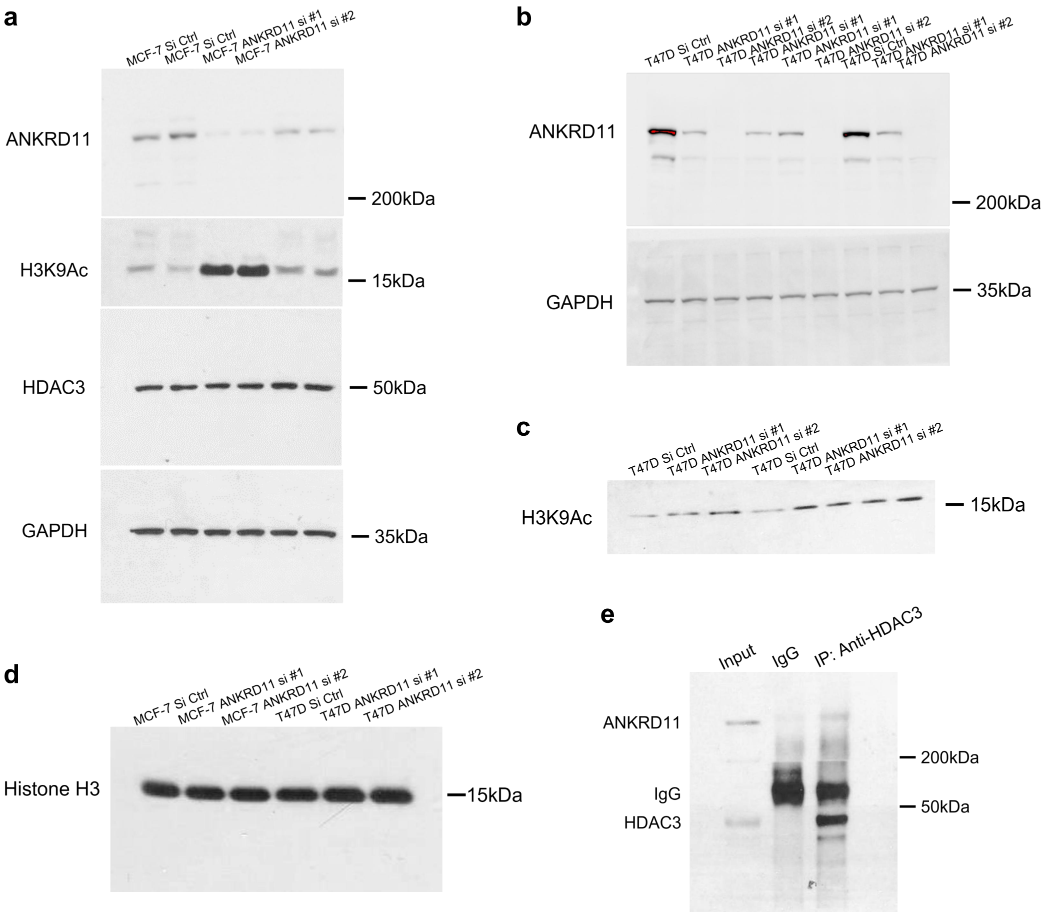

Supplementary Figure 7 The original uncropped images of western blotting of (a) ANKRD11, acetylated histone H3K9, histone H3, HDAC3 and GAPDH in Fig. 5b in MCF-7 cells; (b, c and d) ANKRD11, acetylated histone H3K9, histone H3, HDAC3 and GAPDH in Fig. 5b in T47D cells; (e) ANKRD11 and HDAC3 in anti-HDAC3 antibody precipitates.
