# Supplementary material for: SERPINA3-ANKRD11-HDAC3 pathway induced aromatase inhibitor resistance in breast cancer can be reversed by HDAC3 inhibition
Source: Commun Biol. 2023 Jul 6;6:695. doi: 10.1038/s42003-023-05065-w (PMC10326080; doi:10.1038/s42003-023-05065-w)
Supplement: Supplementary file 3 — Description of Additional Supplementary Files [file 42003_2023_5065_MOESM3_ESM.pdf]

Description of Additional Supplementary Files

File name: Supplementary Data 1
Description: Baseline characteristics of the 1057 breast cancer patients used to evaluate prognostic value of SERPINA3 and ANKRD11 on overall survival.

File name: Supplementary Data 2
Description: The patient populations included for prognostic and expression analysis in GenExMiner v4.9 database.

File name: Supplementary Data 3
Description: The numerical data that makes up the bar graphs in this study.
