# Supplementary material for: SERPINA3-ANKRD11-HDAC3 pathway induced aromatase inhibitor resistance in breast cancer can be reversed by HDAC3 inhibition
Source: Commun Biol. 2023 Jul 6;6:695. doi: 10.1038/s42003-023-05065-w (PMC10326080; doi:10.1038/s42003-023-05065-w)
Supplement: Supplementary file 7 — Reporting Summary [file 42003_2023_5065_MOESM7_ESM.pdf]

nature portfolio

Corresponding author(s): Qiang Liu

Last updated by author(s): May 30, 2023

Reporting Summary

Nature Portfolio wishes to improve the reproducibility of the work that we publish. This form provides structure for consistency and transparency in reporting. For further information on Nature Portfolio policies, see our Editorial Policies and the Editorial Policy Checklist.

Statistics

For all statistical analyses, confirm that the following items are present in the figure legend, table legend, main text, or Methods section.

n/a	Confirmed	
☐	☒	The exact sample size (n) for each experimental group/condition, given as a discrete number and unit of measurement
☐	☒	A statement on whether measurements were taken from distinct samples or whether the same sample was measured repeatedly
☐	☒	The statistical test(s) used AND whether they are one- or two-sided<br>*Only common tests should be described solely by name; describe more complex techniques in the Methods section.*
☐	☒	A description of all covariates tested
☐	☒	A description of any assumptions or corrections, such as tests of normality and adjustment for multiple comparisons
☐	☒	A full description of the statistical parameters including central tendency (e.g. means) or other basic estimates (e.g. regression coefficient) AND variation (e.g. standard deviation) or associated estimates of uncertainty (e.g. confidence intervals)
☐	☒	For null hypothesis testing, the test statistic (e.g. F, t, r) with confidence intervals, effect sizes, degrees of freedom and P value noted<br>*Give P values as exact values whenever suitable.*
☐	☒	For Bayesian analysis, information on the choice of priors and Markov chain Monte Carlo settings
☐	☒	For hierarchical and complex designs, identification of the appropriate level for tests and full reporting of outcomes
☐	☒	Estimates of effect sizes (e.g. Cohen's d, Pearson's r), indicating how they were calculated

Our web collection on statistics for biologists contains articles on many of the points above.

Software and code

Policy information about availability of computer code

Data collection	Gene array analyses identified 366 differentially expressed genes (DEGs) between MCF-7/S0.5 and its tamoxifen resistant (TAMR) cell lines were obtained from a published Elias D's study. DEGs between luminal BC xenografts HBCx22, HBCx34 and their TAMR or ovariectomy resistant (OVAR) derivatives were obtained from published Cottu P's study. The expression profiling data of GSE105777, GSE15347 and GSE147271 were obtained from the Gene Expression Omnibus (GEO) database. Expression profiling dada of 4,861 patients from different studies were included for prognostic analysis by using GenExMiner v4.9 database. Microarray data of 1,057 breast tumors was retrieved from TCGA database. Gene expression correlations were analyzed by using publicly available online databases Molecular Taxonomy of Breast Cancer International Consortium (METABRIC) in cBioPortal.
Data analysis	Downloaded expression microarray data of clinical samples from GEO database were quantile normalized and log2 transformed using R project. Subsequent graphs and differences analyses among groups were performed using Graphpad Prism v9.3.0 with paired Student's t test. Kaplan-Meier survival curves were graphed using Sangerbox online tool, differences were examined using log-rank test. Western blot signal was quantified by image J. Data are presented as mean ± SD in Origin 2019. Differences between samples were evaluated by Student's t-test (two groups) using Graphpad Prism v9.3.0.

For manuscripts utilizing custom algorithms or software that are central to the research but not yet described in published literature, software must be made available to editors and reviewers. We strongly encourage code deposition in a community repository (e.g. GitHub). See the Nature Portfolio guidelines for submitting code & software for further information.

Data

Policy information about availability of data

All manuscripts must include a data availability statement. This statement should provide the following information, where applicable:

- Accession codes, unique identifiers, or web links for publicly available datasets
- A description of any restrictions on data availability
- For clinical datasets or third party data, please ensure that the statement adheres to our policy

RNAseq data generated in this study were deposited in Figshare database with doi: 10.6084/m9.figshare.21786071 and included in supporting data (Supplementary Table S3). Uncropped western blot images are available in Supplementary Fig. S6 and S7.

Human research participants

Policy information about studies involving human research participants and Sex and Gender in Research.

Reporting on sex and gender	N/A
Population characteristics	N/A
Recruitment	N/A
Ethics oversight	N/A

Note that full information on the approval of the study protocol must also be provided in the manuscript.

Field-specific reporting

Please select the one below that is the best fit for your research. If you are not sure, read the appropriate sections before making your selection.

☒ Life sciences ☐ Behavioural & social sciences ☐ Ecological, evolutionary & environmental sciences

For a reference copy of the document with all sections, see nature.com/documents/nr-reporting-summary-flat.pdf

Life sciences study design

All studies must disclose on these points even when the disclosure is negative.

Sample size	No animals or human participants were involved in this study.
Data exclusions	No data were excluded.
Replication	All experiments were reproduced to support conclusions stated in the manuscript.
Randomization	No animals or other objects need to be divided into experimental groups in this research.
Blinding	No animals or other objects need to be divided into experimental groups in this research.

Reporting for specific materials, systems and methods

We require information from authors about some types of materials, experimental systems and methods used in many studies. Here, indicate whether each material, system or method listed is relevant to your study. If you are not sure if a list item applies to your research, read the appropriate section before selecting a response.

Materials & experimental systems

n/a	Involved in the study
☐	☒ Antibodies
☐	☒ Eukaryotic cell lines
☒	☐ Palaeontology and archaeology
☒	☐ Animals and other organisms
☒	☐ Clinical data
☒	☐ Dual use research of concern

Methods

n/a	Involved in the study
☒	☐ ChIP-seq
☒	☐ Flow cytometry
☒	☐ MRI-based neuroimaging

Antibodies

Antibodies used	Anti-SERPINA3 (Huabio, China), anti-ANKRD11 (Cusabio, China), anti-HDAC3 (Huabio, China), anti-acetylated H3K9 (Beyotime, China), anti-histone H3 (Cell Signaling, USA), anti ERα (Cell Signaling, USA), anti-GAPDH (Proteintech, USA, cat.n.:HRP-60004)
Validation	Antibodies critical for novel conclusions were validated by elimination of signals upon KD experiments and/or by functional assays.

Eukaryotic cell lines

Policy information about cell lines and Sex and Gender in Research

Cell line source(s)	Human ER+ BC cell lines MCF-7 and T47D were obtained from the American Type Culture Collection (Manassas, VA, USA). The TAMR and LTED sub-lines were constructed from MCF-7 and T47D respectively in our lab.
Authentication	Cell lines MCF-7 and T47D were authenticated using short tandem repeats analysis.
Mycoplasma contamination	All cell lines were tested for mycoplasma contamination.
Commonly misidentified lines (See ICLAC register)	None of the used cell lines is listed in ICLAC database.
